# The prognostic value of changes in Ki67 following neoadjuvant chemotherapy in residual triple-negative breast cancer: a Swedish nationwide registry-based study

**DOI:** 10.1007/s10549-025-07610-z

**Published:** 2025-01-12

**Authors:** Jenny Nyqvist-Streng, Mikael Helou, Khalil Helou, Chaido Chamalidou, Anikó Kovács, Toshima Z. Parris

**Affiliations:** 1 Department of Surgery, Region Västra Götaland, Skaraborg Hospital, Skövde, Sweden; 2 Department of Surgery, Institute of Clinical Sciences, Sahlgrenska Academy, University of Gothenburg, Gothenburg, Sweden; 3 Department of Psychiatry, Region Västra Götaland, Sahlgrenska University Hospital, Gothenburg, Sweden; 4 Department of Oncology, Institute of Clinical Sciences, Sahlgrenska Academy, University of Gothenburg, Gothenburg, Sweden; 5 Sahlgrenska Center for Cancer Research, Sahlgrenska Academy, University of Gothenburg, Gothenburg, Sweden; 6 Department of Oncology, Region Västra Götaland, Skaraborg Hospital, Skövde, Sweden; 7 Department of Clinical Pathology, Region Västra Götaland, Sahlgrenska University Hospital, Gothenburg, Sweden

**Keywords:** Proliferation marker, Preoperative treatment, Prognosis, Breast cancer, Triple-negative

## Abstract

**Purpose:**

To evaluate the prognostic significance of changes in pre- and post-neoadjuvant chemotherapy (NACT) Ki67 in patients with primary invasive triple-negative breast cancer (TNBC).

**Methods:**

Population-based registry data were retrieved for patients diagnosed with TNBC between 2007 and 2021 (n = 9262). Multivariable Cox regression analysis was performed for disease-specific survival (DSS) and overall survival (OS) adjusted for age and residual disease in the breast and nodes (RDBN).

**Results:**

Of the 1777 TNBC patients receiving NACT, 54 achieved pathologic complete response (pCR) and 755 had residual disease. Most patients were overweight with stage II disease (78%), grade 3 tumors (53%), and RDBN score 3 (42%). Compared to baseline, tumor size (30 *vs.* 15 mm; *P* < 0.0001) and Ki67 levels (63% *vs.* 48%; *P* < 2.2e − 16) generally decreased after NACT. Although only 5% of samples increased in size, Ki67 levels often remained unchanged (75%) or increased (0.9%) after treatment, respectively. However, 34% of patients discontinued treatment. Patients showing no changes in Ki67% had more unfavorable OS (*P* < 0.0001) and DSS (*P* = 0.00032), with significantly lower 5-year survival probabilities (OS: 66%; DSS: 78%) than those with decreased Ki67% (OS: 87%; DSS: 89%). All patients reaching pCR were alive 5 years after diagnosis. However, only the RDBN score was an independent predictor of survival in the multivariable analyses.

**Conclusion:**

Ki67 often remained unchanged in TNBC patients treated with neoadjuvant chemotherapy, resulting in adverse clinical outcomes. These findings highlight the need for individualized treatment regimens and dynamic monitoring of TNBC patients with high Ki67 post-NACT.

**Supplementary Information:**

The online version contains supplementary material available at 10.1007/s10549-025-07610-z.

## Background

Between 10 and 15% of breast tumors are classified as triple-negative breast cancer (TNBC), characterized by negative expression of the estrogen- (ER), progesterone- (PR), and human epidermal growth factor receptor 2 (HER2) receptors. In contrast to hormone-receptor positive breast tumors, TNBCs display aggressive tumor behavior, high proliferation (Ki67), higher risk of relapse and metastatic disease, shorter overall survival, and sensitivity to chemotherapy [1–6]. Ki67s role as a predictive and prognostic marker for breast cancer has been studied extensively [5, 7, 8]. Given the conflicting perspectives regarding Ki67 in breast cancer, further research is warranted to identify the optimal Ki67 threshold associated with prognosis and chemotherapy recommendations [9]. As a crucial proliferation marker in breast cancer, Ki67 expression correlates with response to chemotherapy and clinical outcome [4]. While over 75% of TNBCs show a Ki67 proliferation index exceeding 50% [10–12], some histologic subtypes of TNBC (secretory, low-grade adenosquamous, adenoid cystic) express lower levels of Ki67 and are associated with favorable prognosis [10, 11, 13, 14]. Nevertheless, Ki67 values > 40% are indicative of a higher risk of recurrence and death [12].

Given their sensitivity to chemotherapy and the lack of other treatment choices, patients with TNBC often undergo neoadjuvant and/or adjuvant chemotherapy [15]. Ki67, recognized as an independent predictive factor for neoadjuvant chemotherapy (NACT) efficacy, raises questions about its association with pathologic complete response (pCR) in TNBC [1, 2, 16–21]. Discrepancies persist regarding whether high Ki67 predicts pCR, leading to varied opinions on the need for vigilant follow-up of TNBC patients with high Ki67 post-NACT [22]. Matsubara et al*.* showed that changes in post-NACT Ki67 was an independent prognostic factor in several breast cancer subtypes (Luminal B, TNBC, and HER2 subtypes) [23].

Considering the significance of Ki67 in therapeutic decision-making and implications for choice of treatment and follow-up strategies, further studies are warranted to determine standardized Ki67 thresholds and assess its clinical utility as a predictive and prognostic marker in TNBC, especially in neoadjuvant settings [4, 5, 7, 8, 16, 17, 24, 25]. Using nationwide registry data for 809 patients with primary invasive TNBC (54 reaching pCR and 755 with residual disease, RD), we therefore evaluated the effect of changes in Ki67 levels between baseline and post-NACT on clinical outcome.

## Methods

### Study population and data

**Fig. 1:**
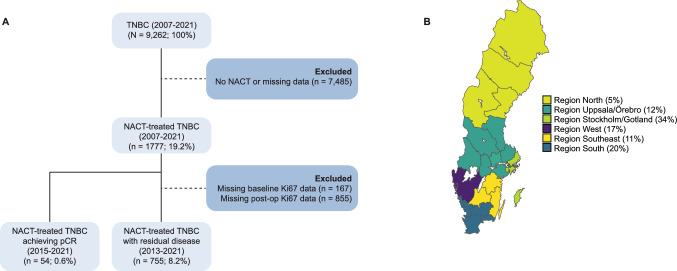
Study overview. **A** Flowchart of triple-negative breast cancer (TNBC) patient selection using Swedish registry data from 2007 to 2021. Of the 9262 patients diagnosed with TNBC during this time period, 1777 received neoadjuvant chemotherapy (NACT), with 54/1777 achieving pathologic complete response and 755/1777 with residual disease and complete data for Ki67 levels at baseline and post-NACT. **B** Map of Sweden showing the Swedish healthcare regions where the 809 patients were treated. The percentage of patients treated in each region is shown in parentheses

In this retrospective study, population-based registry data for 9,262 women diagnosed with TNBC (either at baseline or after surgery) between 2007 and 2021 were retrieved from several Swedish registers, i.e., National Breast Cancer Register (NBCR), Swedish Patient Register (SPR), and Swedish Cause of Death Register (SCDR). NBCR provided data for the primary tumor (e.g., tumor stage, axillary lymph node status), patient (e.g., age, menopausal status), and treatment, while data on comorbidities were retrieved from SPR and cause of death from SCDR. International classification of disease (ICD) codes for different diseases (i.e., myocardial infarction, congestive heart failure, peripheral vascular disease, cerebrovascular disease, pulmonary diseases, rheumatic disease, dementia, hemiplegia, diabetes, chronic kidney disease, liver disease, gastric peptic ulcer disease, cancer, and HIV/AIDS) were retrieved from SPR (in- and outpatient data) to calculate the weighted Charlson comorbidity index (CCIw; a measure of the burden of comorbidities), as described elsewhere [26]. All procedures were done in accordance with the Declaration of Helsinki and approved by the Central Ethical Review Board and the Swedish Ethical Review Authority (reference numbers 2019–05676; 2021–04421; 2022–02946-02; 2023-07905-02). A flowchart of the patient selection process is shown in Fig. 1A.

### Outcomes and definitions

To determine the effect of changes in Ki67 on survival outcomes for patients receiving NACT, patients with incomplete data for NACT (n = 7485) or Ki67 at baseline (n = 167) and post-NACT (n = 855) were excluded. Pathological staging after NACT (ypTNM) was based on AJCC cancer staging [27]. Of the 1777 patients that received NACT, 54 achieved pCR, defined as the absence of invasive cancer (and only cancer in situ) in the breast (ypT0/Tis) following NACT. Residual disease was stratified by Residual Disease in Breast and Nodes (RDBN) and region (local and regional). The RDBN score (1–4) was calculated a formula for the RDBN index (0 to ≥ 4.4): RDBN = 0.2 × tumor size post-NACT (cm) + lymph node stage post-NACT (0–3) + histologic grade post-NACT (1–3), with RDBN score 1 corresponding to pCR in the breast and nodes (RDBN index 0), RDBN score 2 as residual carcinoma (RDBN index 0.1–2.9), RDBN score 3 as residual carcinoma (RDBN index 3.0–4.3), and RDBN score 4 as residual carcinoma (RDBN index ≥ 4.4) [28]. For bilateral breast cancer cases, only the first diagnosed tumor was included.

Cases with Ki67 < 20% were classified as low Ki67 expression, while ≥ 20% was used to classify samples with high Ki67 expression, according to national guidelines and equivalent to the 2017 St. Gallen molecular classification for Ki67 [29]. A Ki67 ratio was calculated by dividing Ki67% at baseline by Ki67% post-NACT. Changes in Ki67 were defined as increased if Ki67 changed from low (baseline) to high (post-NACT), decreased if Ki67 changed from high (baseline) to low (post-NACT), and no changes if Ki67 remained the same between baseline and post-NACT. The receptor-based subtypes (luminal A, luminal B HER2-negative, luminal B HER2-positive, non-luminal HER2-positive, TNBC) were determined using Swedish National guidelines, i.e. luminal A: ER + , PR ± , HER2-, Ki67 low; luminal B HER2-negative: ER + , PR ± , HER2-, Ki67 high; luminal B HER2-positive: ER + , PR ± , HER2 + , any Ki67; non-luminal HER2-positive: ER-, PR-, HER2 + ; TNBC: ER-, PR-, HER2- [30].

### Statistical analysis

Statistical analyses were performed using R/Bioconductor (version 4.2.2) with a significance threshold set at 0.05 and two-sided p-values. Descriptive statistics and Sankey plots were done using the tableone (version 0.13.2)[31] and alluvial packages (version 0.1–2) in R, respectively [32]. P-values were calculated using Chi-square test for categorical variables (with continuity correction) and ANOVA for continuous variables with the tableone script. Survival rates were depicted with Kaplan–Meier curves and tested with log-rank test using the ggsurvfit R package (version 0.3.0) [33]. Cumulative incidence curves for competing risks were constructed using ggcompetingrisks function in the survminer package (version 0.4.9) in R. Univariable Cox proportional hazards models were calculated for decreased changes or no changes in Ki67 levels at baseline and post-NACT using the survival R package (version 3.4–0); cases with increased Ki67 were removed from the analysis. Multivariable models were adjusted for patient age and RDBN score. Patient survival rates were defined as the time from initial diagnosis to breast cancer-related death for disease-specific survival (DSS) and the time from initial diagnosis to death from any cause for overall survival (OS).

## Results

### Patient characteristics

Of the 9,262 patients diagnosed with TNBC in Sweden between 2007 and 2021, 1777 received neoadjuvant chemotherapy. In total, 54/1777 patients achieving pCR and 755/1777 with RD and complete data for Ki67% at baseline and post-NACT were included in the study (all diagnosed between 2013 and 2021; Table 1 and Fig. 1A). One-third (34%) of the 809 patients were diagnosed in the Stockholm/Gotland healthcare region, while only 5% originated from the North healthcare region (Fig. 1B). The median age at baseline was 54 years (IQR, 43–65), with 74% of patients under 65 years of age. The majority of patients (87%) had stage I-II disease with no evidence of axillary lymph node metastases (58%) or distant metastases (100%). Furthermore, most patients received mastectomy/breast-conserving surgery and adjuvant radiotherapy (75%), and either sentinel node (SN; 48%) or axillary lymph node dissection (ALND; 42%). High Ki67 was more prevalent in the pCR group (96% for pCR vs. 94% for residual disease; *P* < 0.001) and only one tumor sample in the RD group was classified as non-TNBC (HER2 +) at baseline. No difference in any of the other variables at baseline was found between patients reaching pCR and those with RD.

**Table 1:** Clinicopathological characteristics of the 809 patients with TNBC treated with neoadjuvant chemotherapy, stratified by pCR and RD

Characteristic	Overall (n = 809)	Pathologic complete response (n = 54)	Residual disease (n = 755)	*P*
Swedish region (%)				0.235
North	44 (5.4)	2 (3.7)	42 (5.6)	
Stockholm/Gotland	276 (34.1)	15 (27.8)	261 (34.6)	
South	163 (20.1)	18 (33.3)	145 (19.2)	
Southeast	91 (11.2)	4 (7.4)	87 (11.5)	
Uppsala/Örebro	99 (12.2)	6 (11.1)	93 (12.3)	
West	136 (16.8)	9 (16.7)	127 (16.8)	
Patient age at baseline (median [IQR])	54.00 [43.00, 65.00]	51.50 [42.00, 61.00]	54.00 [43.00, 65.00]	0.312
Age range at baseline (%)				0.611
< 40	142 (17.6)	9 (16.7)	133 (17.6)	
40–49	192 (23.7)	17 (31.5)	175 (23.2)	
50–64	266 (32.9)	18 (33.3)	248 (32.8)	
65–74	146 (18.0)	7 (13.0)	139 (18.4)	
≥ 75	63 (7.8)	3 (5.6)	60 (7.9)	
Stage at baseline (%)				0.52
IA	80 (9.9)	9 (16.7)	71 (9.4)	
IIA	393 (48.6)	24 (44.4)	369 (48.9)	
IIB	233 (28.8)	17 (31.5)	216 (28.6)	
IIIA	68 (8.4)	3 (5.6)	65 (8.6)	
IIIB	13 (1.6)	0 (0.0)	13 (1.7)	
IIIC	13 (1.6)	1 (1.9)	12 (1.6)	
Unspecified	9 (1.1)	0 (0.0)	9 (1.2)	
Tumor size (mm) at baseline (median [IQR])	30.00 [23.00, 40.75]	23.50 [19.50, 40.50]	30.00 [24.00, 40.75]	0.072
Ki67% at baseline (mean (SD))	63.27 (24.27)	67.49 (21.92)	62.98 (24.41)	0.191
Ki67 levels at baseline (%)				** < 0.001**
High	760 (93.9)	52 (96.3)	708 (93.8)	
Low	48 (5.9)	1 (1.9)	47 (6.2)	
Unknown	1 (0.1)	1 (1.9)	0 (0.0)	
Subtype at baseline (%)				1
TNBC	808 (99.9)	54 (100.0)	754 (99.9)	
HER2 +	1 (0.1)	0 (0.0)	1 (0.1)	
Luminal A	0 (0.0)	0 (0.0)	0 (0.0)	
Luminal B/HER2-	0 (0.0)	0 (0.0)	0 (0.0)	
Luminal B/HER2 +	0 (0.0)	0 (0.0)	0 (0.0)	
Breast surgery (%)				
Mx	378 (46.7)	27 (50.0)	351 (46.5)	0.960
BCS	402 (49.7)	25 (46.3)	377 (49.9)	
Subcutaneous Mx	25 (3.1)	2 (3.7)	23 (3.0)	
Only axilla surgery	2 (0.2)	0 (0.0)	2 (0.3)	
Missing data	2 (0.2)	0 (0.0)	2 (0.3)	
Axillary surgery (%)				0.831
SN	389 (48.1)	29 (53.7)	360 (47.7)	
SN + ALND	62 (7.7)	3 (5.6)	59 (7.8)	
ALND	341 (42.2)	21 (38.9)	320 (42.4)	
Missing data	17 (2.1)	1 (1.9)	16 (2.1)	
Locoregional treatment (%)				0.242
BCS + ART	359 (44.4)	22 (40.7)	337 (44.6)	
Mx + ART	250 (30.9)	15 (27.8)	235 (31.1)	
Mx-ART	68 (8.4)	3 (5.6)	65 (8.6)	
Other	132 (16.3)	14 (25.9)	118 (15.6)	
Tumor size (mm) post-NACT (median [IQR])	15.00 [8.00, 27.00]	NA [NA, NA]	15.00 [8.00, 27.00]	NA
Stage post-NACT (ypTNM)				** < 0.001**
0	39 (4.8)	39 (72.2)	0 (0.0)	
IA	273 (33.7)	0 (0.0)	273 (36.2)	
IIA	199 (24.6)	0 (0.0)	199 (26.4)	
IIB	108 (13.3)	0 (0.0)	108 (14.3)	
IIIA	42 (5.2)	0 (0.0)	42 (5.6)	
Unspecified	148 (18.3)	15 (27.8)	133 (17.6)	
Residual disease (%)				** < 0.001**
pCR	54 (6.7)	54 (100.0)	0 (0.0)	
Local residual disease	437 (54.0)	0 (0.0)	437 (57.9)	
Regional residual disease	301 (37.2)	0 (0.0)	301 (39.9)	
Unspecified	17 (2.1)	0 (0.0)	17 (2.3)	
RDBN score (%)				** < 0.001**
1	54 (6.7)	54 (100.0)	0 (0.0)	
2	157 (19.4)	0 (0.0)	157 (20.8)	
3	316 (39.1)	0 (0.0)	316 (41.9)	
4	116 (14.3)	0 (0.0)	116 (15.4)	
Missing data	166 (20.5)	0 (0.0)	166 (22.0)	
NHG post-NACT (%)				** < 0.001**
Grade 1	17 (2.1)	1 (1.9)	16 (2.1)	
Grade 2	270 (33.4)	4 (7.4)	266 (35.2)	
Grade 3	411 (50.8)	13 (24.1)	398 (52.7)	
Not evaluable	80 (9.9)	10 (18.5)	70 (9.3)	
Not performed	8 (1.0)	4 (7.4)	4 (0.5)	
Missing data	23 (2.8)	22 (40.7)	1 (0.1)	
Ki67% post-NACT (mean (SD))	48.40 (32.90)	NaN (NA)	48.40 (32.90)	NA
Ki67 levels post-NACT (%)				** < 0.001**
High	534 (66.0)	0 (0.0)	534 (70.7)	
Low	221 (27.3)	0 (0.0)	221 (29.3)	
Not applicable	54 (6.7)	54 (100.0)	0 (0.0)	
Subtype post-NACT (%)				** < 0.001**
TNBC	672 (83.1)	0 (0.0)	672 (89.0)	
HER2 +	13 (1.6)	0 (0.0)	13 (1.7)	
Luminal A	13 (1.6)	0 (0.0)	13 (1.7)	
Luminal B/HER2-	25 (3.1)	0 (0.0)	25 (3.3)	
Luminal B/HER2 +	0 (0.0)	0 (0.0)	0 (0.0)	
Not applicable	54 (6.7)	54 (100.0)	0 (0.0)	
Not determined	32 (4.0)	0 (0.0)	32 (4.2)	
Ki67 ratio^a^ (mean (SD))	3.34 (6.27)	NaN (NA)	3.34 (6.27)	NA
Adjuvant chemotherapy (%)				** < 0.001**
Yes	377 (46.6)	7 (13.0)	370 (49.0)	
No	336 (41.5)	34 (63.0)	302 (40.0)	
Missing data	96 (11.9)	13 (24.1)	83 (11.0)	
Adjuvant radiotherapy (%)				**0.014**
Yes	624 (77.1)	37 (68.5)	587 (77.7)	
No	89 (11.0)	4 (7.4)	85 (11.3)	
Missing data	96 (11.9)	13 (24.1)	83 (11.0)	
Adjuvant bisphosphonate (%)				**0.02**
Yes	201 (24.8)	12 (22.2)	189 (25.0)	
No	510 (63.0)	29 (53.7)	481 (63.7)	
Missing data	98 (12.1)	13 (24.1)	85 (11.3)	
Charlson comorbidity index, weighted (%)				0.858
0	636 (78.6)	47 (87.0)	589 (78.0)	
1	70 (8.7)	3 (5.6)	67 (8.9)	
2	84 (10.4)	3 (5.6)	81 (10.7)	
3	10 (1.2)	1 (1.9)	9 (1.2)	
4	3 (0.4)	0 (0.0)	3 (0.4)	
5	3 (0.4)	0 (0.0)	3 (0.4)	
6	2 (0.2)	0 (0.0)	2 (0.3)	
8	1 (0.1)	0 (0.0)	1 (0.1)	

Statistically significant p-values are highlighted in bold

*ART* Adjuvant radiotherapy, *BCS* Breast-conserving surgery, *Mx* Mastectomy, *NA* Not applicable, *NACT* Neoadjuvant chemotherapy, *NHG* Nottingham grade, *pCR* Pathologic complete response, *RD* Residual disease, *RDBN* Residual disease in breast and nodes, *TNBC* Triple-negative breast cancer

P-values were calculated using Chi-square test for categorical variables (with continuity correction) and ANOVA for continuous variables

^a^Ki67 ratio = Ki67% at baseline / Ki67% post-NACT

The 809 patients primarily received NACT with anthracyclines and a taxane (Table 2). Capecitabine use was more common among patients achieving pCR. Approximately 66% of patients completed NACT according to plan, with side effects to treatment reported as the most common reason for treatment discontinuation and less than 20% of patients were admitted to in-patient care due to complications. Patients with RD more frequently received adjuvant treatment (adjuvant chemotherapy: 49% RD vs 13% pCR; adjuvant radiotherapy: 78% RD vs 69% pCR; adjuvant bisphosphonate: 25% RD vs 22% pCR; Table 1). Moreover, local residual disease (58%) and RDBN score 3 (42%) were prevalent, followed by RDBN scores 2 (21%) and 4 (15%).

**Table 2:** Type of neoadjuvant treatment the 809 TNBC patients received, stratified by pCR and changes in Ki67 levels at baseline and post-NACT

Characteristic	Overall (n = 809)	Decreased Ki67 levels (n = 181)	Increased Ki67 levels (n = 7)	No changes (n = 567)	Pathologic complete response (n = 54)	*P*
Type of NACT (%)						0.172
Anthracycline + taxane	720 (89.0)	170 (93.9)	7 (100.0)	491 (86.6)	52 (96.3)	
Anthracycline-based	40 (4.9)	5 (2.8)	0 (0.0)	35 (6.2)	0 (0.0)	
Taxane-based	45 (5.6)	6 (3.3)	0 (0.0)	37 (6.5)	2 (3.7)	
Other	4 (0.5)	0 (0.0)	0 (0.0)	4 (0.7)	0 (0.0)	
Anthracycline (%)						0.192
Yes	760 (93.9)	175 (96.7)	7 (100.0)	526 (92.8)	52 (96.3)	
Missing data	49 (6.1)	6 (3.3)	0 (0.0)	41 (7.2)	2 (3.7)	
Docetaxel (%)						0.519
Yes	374 (46.2)	82 (45.3)	3 (42.9)	269 (47.4)	20 (37.0)	
Missing data	435 (53.8)	99 (54.7)	4 (57.1)	298 (52.6)	34 (63.0)	
Paclitaxel (%)						0.098
Yes	421 (52.0)	99 (54.7)	5 (71.4)	282 (49.7)	35 (64.8)	
Missing data	388 (48.0)	82 (45.3)	2 (28.6)	285 (50.3)	19 (35.2)	
Capecitabine (%)						**0.026**
Yes	33 (4.1)	12 (6.6)	0 (0.0)	16 (2.8)	5 (9.3)	
Missing data	776 (95.9)	169 (93.4)	7 (100.0)	551 (97.2)	49 (90.7)	
Other (%)						0.093
Yes	230 (28.4)	62 (34.3)	1 (14.3)	148 (26.1)	19 (35.2)	
Missing data	579 (71.6)	119 (65.7)	6 (85.7)	419 (73.9)	35 (64.8)	
Completed treatment according to plan (%)						0.514
Yes	536 (66.3)	118 (65.2)	4 (57.1)	371 (65.4)	43 (79.6)	
No	272 (33.6)	63 (34.8)	3 (42.9)	195 (34.4)	11 (20.4)	
Missing data	1 (0.1)	0 (0.0)	0 (0.0)	1 (0.2)	0 (0.0)	
Reason for discontinuation of treatment (%)						0.117
Side effects	201 (24.8)	51 (28.2)	3 (42.9)	137 (24.2)	10 (18.5)	
Other	71 (8.8)	12 (6.6)	0 (0.0)	58 (10.2)	1 (1.9)	
Missing data	537 (66.4)	118 (65.2)	4 (57.1)	372 (65.6)	43 (79.6)	
In-patient care due to complications (%)						0.692
Yes	146 (18.0)	32 (17.7)	0 (0.0)	106 (18.7)	8 (14.8)	
No	659 (81.5)	149 (82.3)	7 (100.0)	457 (80.6)	46 (85.2)	
Missing data	4 (0.5)	0 (0.0)	0 (0.0)	4 (0.7)	0 (0.0)	

Statistically significant p-values are highlighted in bold

*NACT* Neoadjuvant chemotherapy, *pCR* Pathologic complete response, *TNBC* Triple-negative breast cancer

P-values were calculated using Chi-square test for categorical variables (with continuity correction) and ANOVA for continuous variables

### Effect of NACT on tumor characteristics

**Fig. 2:**
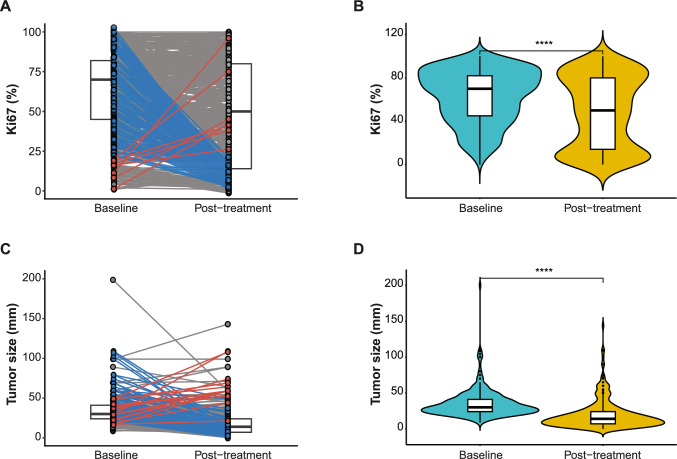
Before-after plots for **A**, **B** Ki67 (%) and **C**, **D** tumor size (mm) at baseline and post-treatment (neoadjuvant chemotherapy) in the 755 patients with residual disease. Gray, blue, and red colored lines depict no changes, decreased, and increased **A** Ki67 levels or **C** tumor size post-treatment, respectively. At baseline, high Ki67 expression (Ki67 ≥ 20%) was prevalent among TNBC. Although tumor size and Ki67 levels were generally lower following treatment, Ki67 remained high in the surgical specimens of most TNBCs. T-test was used to calculate statistically significant differences in Ki67% and tumor size between baseline and post-treatment. *P < 0.05; **P ≤ 0.01; ***P ≤ 0.001; ****P ≤ 0.0001

**Fig. 3:**
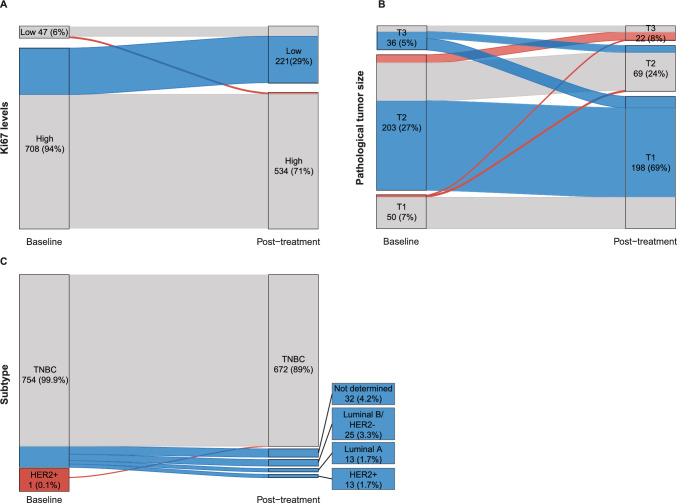
Sankey plots showing changes in **A** Ki67 levels, **B** tumor size, and **C** subtype for tumor samples at baseline and post-treatment in the 755 patients with residual disease. Gray depicts samples with no changes in Ki67, pathological tumor size or breast cancer subtype, while blue and red depict a decrease and increase in status, respectively. At baseline, the majority of patients with residual disease had cT2 tumors classified as TNBC with high Ki67 expression. After neoadjuvant chemotherapy, subtype changes from TNBC to HER2 + , Luminal A, and Luminal B/HER2- were common. In addition, the tumors were predominantly smaller but still had high Ki67 expression

In patients with residual disease, Ki67% and tumor size were significantly lower post-NACT (*P* < 0.0001; Fig. 2). However, 71% of tumors still showed high Ki67 levels with > 20% expression after treatment (527 samples with high and 7 with low Ki67 at baseline; Fig. 3A). Although relatively few tumors were larger after NACT (3 cT1 tumors at baseline were cT2 and cT3 after treatment and 12 cT2 at baseline were cT3 after NACT), pathological tumor size did not change after treatment for 113 TNBCs (15%; Fig. 3B). At baseline, one of the 755 tumors was classified as HER2 + (0.1%). After NACT, only 89% of the samples were classified as TNBC, with the remaining 11% classified as Luminal A (2%), Luminal B/HER2- (3%), HER2 + (2%) or Not determined (4%; Fig. 3C). In total, 181 (24%), 7 (0.9%), and 567 cases (75%) were determined to have decreased, increased, and no changes in Ki67 levels following NACT, respectively (Table 3). On average, the Ki67 ratio was 11.1 (SD, 10.3) for decreased Ki67%, 0.3 (SD, 0.2) for increased Ki67%, and 1.3 (SD, 0.8) for no changes in Ki67%. The absence of changes in Ki67 levels was more prevalent in stage III (*P* = 0.005), NHG grade III (*P* < 0.001) tumors of intermediate tumor size (median, 16 mm [IQR, 8–30]; *P* < 0.001) and mean Ki67% around 62% (SD, 27%; *P* < 0.001) following treatment. Furthermore, significantly more patients with decreased Ki67 levels were treated with breast-conserving surgery and adjuvant radiotherapy.

**Table 3:** Clinicopathological characteristics of the 809 patients with TNBC treated with neoadjuvant chemotherapy, stratified by pCR and changes in Ki67 levels at baseline and post-NACT

Characteristic	Overall (n = 809)	Decreased Ki67 levels (n = 181)	Increased Ki67 levels (n = 7)	No changes (n = 567)	Pathologic complete response (n = 54)	*P*
Swedish region (%)						** < 0.001**
North	44 (5.4)	1 (0.6)	0 (0.0)	41 (7.2)	2 (3.7)	
Stockholm/Gotland	276 (34.1)	67 (37.0)	1 (14.3)	193 (34.0)	15 (27.8)	
South	163 (20.1)	44 (24.3)	0 (0.0)	101 (17.8)	18 (33.3)	
Southeast	91 (11.2)	11 (6.1)	1 (14.3)	75 (13.2)	4 (7.4)	
Uppsala/Örebro	99 (12.2)	26 (14.4)	0 (0.0)	67 (11.8)	6 (11.1)	
West	136 (16.8)	32 (17.7)	5 (71.4)	90 (15.9)	9 (16.7)	
Patient age at baseline (median [IQR])	54.00 [43.00, 65.00]	56.00 [46.00, 65.00]	55.00 [48.50, 66.00]	53.00 [42.00, 65.00]	51.50 [42.00, 61.00]	0.296
Age range at baseline (%)						0.488
< 40	142 (17.6)	23 (12.7)	1 (14.3)	109 (19.2)	9 (16.7)	
40–49	192 (23.7)	39 (21.5)	1 (14.3)	135 (23.8)	17 (31.5)	
50–64	266 (32.9)	73 (40.3)	2 (28.6)	173 (30.5)	18 (33.3)	
65–74	146 (18.0)	32 (17.7)	2 (28.6)	105 (18.5)	7 (13.0)	
≥ 75	63 (7.8)	14 (7.7)	1 (14.3)	45 (7.9)	3 (5.6)	
Stage at baseline (%)						**0.018**
IA	80 (9.9)	29 (16.0)	0 (0.0)	42 (7.4)	9 (16.7)	
IIA	393 (48.6)	84 (46.4)	4 (57.1)	281 (49.6)	24 (44.4)	
IIB	233 (28.8)	50 (27.6)	2 (28.6)	164 (28.9)	17 (31.5)	
IIIA	68 (8.4)	13 (7.2)	0 (0.0)	52 (9.2)	3 (5.6)	
IIIB	13 (1.6)	1 (0.6)	0 (0.0)	12 (2.1)	0 (0.0)	
IIIC	13 (1.6)	0 (0.0)	1 (14.3)	11 (1.9)	1 (1.9)	
Unspecified	9 (1.1)	4 (2.2)	0 (0.0)	5 (0.9)	0 (0.0)	
Tumor size at baseline (median [IQR])	30.00 [23.00, 40.75]	30.00 [23.00, 40.00]	30.00 [27.75, 31.25]	30.00 [25.00, 42.00]	23.50 [19.50, 40.50]	0.185
Ki67% at baseline (mean (SD))	63.27 (24.27)	58.57 (23.18)	12.14 (6.87)	65.01 (24.08)	67.49 (21.92)	** < 0.001**
Ki67 levels at baseline (%)						** < 0.001**
High	760 (93.9)	181 (100.0)	0 (0.0)	527 (92.9)	52 (96.3)	
Low	48 (5.9)	0 (0.0)	7 (100.0)	40 (7.1)	1 (1.9)	
Unknown	1 (0.1)	0 (0.0)	0 (0.0)	0 (0.0)	1 (1.9)	
Subtype at baseline (%)						0.935
TNBC	808 (99.9)	181 (100.0)	7 (100.0)	566 (99.8)	54 (100.0)	
HER2 +	1 (0.1)	0 (0.0)	0 (0.0)	1 (0.2)	0 (0.0)	
Luminal A	0 (0.0)	0 (0.0)	0 (0.0)	0 (0.0)	0 (0.0)	
Luminal B/HER2-	0 (0.0)	0 (0.0)	0 (0.0)	0 (0.0)	0 (0.0)	
Luminal B/HER2 +	0 (0.0)	0 (0.0)	0 (0.0)	0 (0.0)	0 (0.0)	
Breast surgery (%)						** < 0.001**
Mx	378 (46.7)	62 (34.3)	3 (42.9)	286 (50.4)	27 (50.0)	
BCS	402 (49.7)	110 (60.8)	3 (42.9)	264 (46.6)	25 (46.3)	
Subcutaneous Mx	25 (3.1)	9 (5.0)	0 (0.0)	14 (2.5)	2 (3.7)	
Only axilla surgery	2 (0.2)	0 (0.0)	0 (0.0)	2 (0.4)	0 (0.0)	
Missing data	2 (0.2)	0 (0.0)	1 (14.3)	1 (0.2)	0 (0.0)	
Axillary surgery (%)						0.134
SN	389 (48.1)	102 (56.4)	3 (42.9)	255 (45.0)	29 (53.7)	
SN + ALND	62 (7.7)	13 (7.2)	0 (0.0)	46 (8.1)	3 (5.6)	
ALND	341 (42.2)	63 (34.8)	3 (42.9)	254 (44.8)	21 (38.9)	
Missing data	17 (2.1)	3 (1.7)	1 (14.3)	12 (2.1)	1 (1.9)	
Locoregional treatment (%)						**0.002**
BCS + ART	359 (44.4)	100 (55.2)	3 (42.9)	234 (41.3)	22 (40.7)	
Mx + ART	250 (30.9)	36 (19.9)	2 (28.6)	197 (34.7)	15 (27.8)	
Mx-ART	68 (8.4)	10 (5.5)	0 (0.0)	55 (9.7)	3 (5.6)	
Other	132 (16.3)	35 (19.3)	2 (28.6)	81 (14.3)	14 (25.9)	
Tumor size post-NACT (median [IQR])	15.00 [8.00, 27.00]	11.00 [6.00, 17.00]	22.00 [16.00, 29.00]	16.00 [8.00, 30.00]	NA [NA, NA]	** < 0.001**
Stage post-NACT (ypTNM)						** < 0.001**
0	39 (4.8)	0 (0.0)	0 (0.0)	0 (0.0)	39 (72.2)	
IA	273 (33.7)	80 (44.2)	1 (14.3)	192 (33.9)	0 (0.0)	
IIA	199 (24.6)	56 (30.9)	3 (42.9)	140 (24.7)	0 (0.0)	
IIB	108 (13.3)	16 (8.8)	2 (28.6)	90 (15.9)	0 (0.0)	
IIIA	42 (5.2)	2 (1.1)	1 (14.3)	39 (6.9)	0 (0.0)	
Unspecified	148 (18.3)	27 (14.9)	0 (0.0)	106 (18.7)	15 (27.8)	
Residual disease (%)						** < 0.001**
Local residual disease	437 (54.0)	116 (64.1)	2 (28.6)	319 (56.3)	0 (0.0)	
Regional residual disease	301 (37.2)	60 (33.1)	5 (71.4)	236 (41.6)	0 (0.0)	
pCR	54 (6.7)	0 (0.0)	0 (0.0)	0 (0.0)	54 (100.0)	
Unspecified	17 (2.1)	5 (2.8)	0 (0.0)	12 (2.1)	0 (0.0)	
RDBN score (%)						** < 0.001**
1	54 (6.7)	0 (0.0)	0 (0.0)	0 (0.0)	54 (100.0)	
2	157 (19.4)	80 (44.2)	0 (0.0)	75 (13.2)	0 (0.0)	
3	316 (39.1)	62 (34.3)	2 (28.6)	252 (44.4)	0 (0.0)	
4	116 (14.3)	3 (1.7)	2 (28.6)	110 (19.4)	0 (0.0)	
Missing data	166 (20.5)	36 (19.9)	3 (42.9)	130 (22.9)	0 (0.0)	
NHG post-NACT (%)						** < 0.001**
Grade 1	17 (2.1)	8 (4.4)	1 (14.3)	7 (1.2)	1 (1.9)	
Grade 2	270 (33.4)	127 (70.2)	3 (42.9)	136 (24.0)	4 (7.4)	
Grade 3	411 (50.8)	20 (11.0)	3 (42.9)	375 (66.1)	13 (24.1)	
Not evaluable	80 (9.9)	24 (13.3)	0 (0.0)	46 (8.1)	10 (18.5)	
Not performed	8 (1.0)	2 (1.1)	0 (0.0)	2 (0.4)	4 (7.4)	
Missing data	23 (2.8)	0 (0.0)	0 (0.0)	1 (0.2)	22 (40.7)	
Ki67% post-NACT (mean (SD))	48.40 (32.90)	7.15 (5.50)	50.86 (24.52)	61.53 (26.63)	NaN (NA)	** < 0.001**
Ki67 levels post-NACT (%)						** < 0.001**
High	534 (66.0)	0 (0.0)	7 (100.0)	527 (92.9)	0 (0.0)	
Low	221 (27.3)	181 (100.0)	0 (0.0)	40 (7.1)	0 (0.0)	
Not applicable	54 (6.7)	0 (0.0)	0 (0.0)	0 (0.0)	54 (100.0)	
Subtype post-NACT (%)						** < 0.001**
TNBC	672 (83.1)	141 (77.9)	7 (100.0)	524 (92.4)	0 (0.0)	
HER2 +	13 (1.6)	1 (0.6)	0 (0.0)	12 (2.1)	0 (0.0)	
Luminal A	13 (1.6)	7 (3.9)	0 (0.0)	6 (1.1)	0 (0.0)	
Luminal B/HER2-	25 (3.1)	12 (6.6)	0 (0.0)	13 (2.3)	0 (0.0)	
Luminal B/HER2 +	0 (0.0)	0 (0.0)	0 (0.0)	0 (0.0)	0 (0.0)	
Not applicable	54 (6.7)	0 (0.0)	0 (0.0)	0 (0.0)	54 (100.0)	
Not determined	32 (4.0)	20 (11.0)	0 (0.0)	12 (2.1)	0 (0.0)	
Ki67 ratio^a^ (mean (SD))	3.34 (6.27)	11.07 (10.33)	0.29 (0.21)	1.26 (0.84)	-	** < 0.001**
Adjuvant chemotherapy (%)						** < 0.001**
Yes	377 (46.6)	68 (37.6)	6 (85.7)	296 (52.2)	7 (13.0)	
No	336 (41.5)	87 (48.1)	0 (0.0)	215 (37.9)	34 (63.0)	
Missing data	96 (11.9)	26 (14.4)	1 (14.3)	56 (9.9)	13 (24.1)	
Adjuvant radiotherapy (%)						**0.032**
Yes	624 (77.1)	140 (77.3)	6 (85.7)	441 (77.8)	37 (68.5)	
No	89 (11.0)	15 (8.3)	0 (0.0)	70 (12.3)	4 (7.4)	
Missing data	96 (11.9)	26 (14.4)	1 (14.3)	56 (9.9)	13 (24.1)	
Adjuvant bisphosphonate (%)						**0.043**
Yes	201 (24.8)	48 (26.5)	0 (0.0)	141 (24.9)	12 (22.2)	
No	510 (63.0)	107 (59.1)	6 (85.7)	368 (64.9)	29 (53.7)	
Missing data	98 (12.1)	26 (14.4)	1 (14.3)	58 (10.2)	13 (24.1)	
Charlson comorbidity index, weighted (%)						0.692
0	636 (78.6)	136 (75.1)	5 (71.4)	448 (79.0)	47 (87.0)	
1	70 (8.7)	10 (5.5)	1 (14.3)	56 (9.9)	3 (5.6)	
2	84 (10.4)	27 (14.9)	1 (14.3)	53 (9.3)	3 (5.6)	
3	10 (1.2)	5 (2.8)	0 (0.0)	4 (0.7)	1 (1.9)	
4	3 (0.4)	1 (0.6)	0 (0.0)	2 (0.4)	0 (0.0)	
5	3 (0.4)	1 (0.6)	0 (0.0)	2 (0.4)	0 (0.0)	
6	2 (0.2)	1 (0.6)	0 (0.0)	1 (0.2)	0 (0.0)	
8	1 (0.1)	0 (0.0)	0 (0.0)	1 (0.2)	0 (0.0)	

Statistically significant p-values are highlighted in bold

*ART* Adjuvant radiotherapy, *BCS* Breast-conserving surgery, *Mx* Mastectomy, *NACT* Neoadjuvant chemotherapy, *NHG* Nottingham grade, *pCR* Pathologic complete response, *RDBN* Residual disease in breast and nodes, *TNBC* Triple-negative breast cancer

P-values were calculated using Chi-square test for categorical variables (with continuity correction) and ANOVA for continuous variables

^a^Ki67 ratio = Ki67% at baseline / Ki67% post-NACT

### Effect of changes in Ki67 levels on clinical outcome

**Fig. 4:**
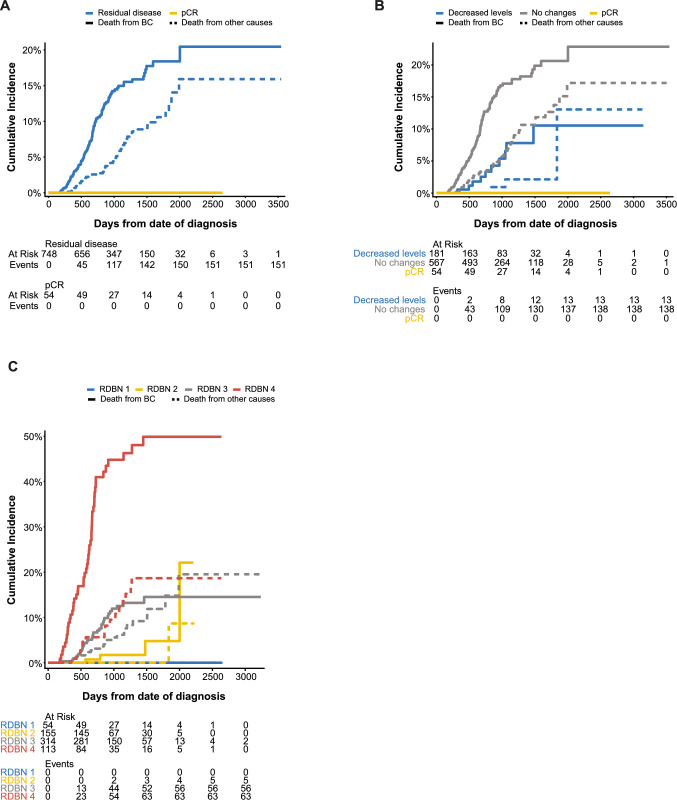
Competing risk survival analysis of death from breast cancer (BC; solid line) or from other causes (dotted line) in **A** TNBC patients with residual disease (blue) or those achieving pathologic complete response (pCR; yellow), **B** patients with decreased Ki67 levels (blue), no changes in Ki67 levels (gray) or pCR (yellow), and **C** patients with residual disease in the breast and nodes (RDBN) score 1 (blue), RDBN score 2 (yellow), RDBN score 3 (gray), and RDBN score 4 (red). Patients with increased Ki67 levels (n = 7) were removed from the analysis. Patients with residual disease, no changes in Ki67 levels post-NACT, and RDBN score 4 had the highest risk of death of BC

**Fig. 5:**
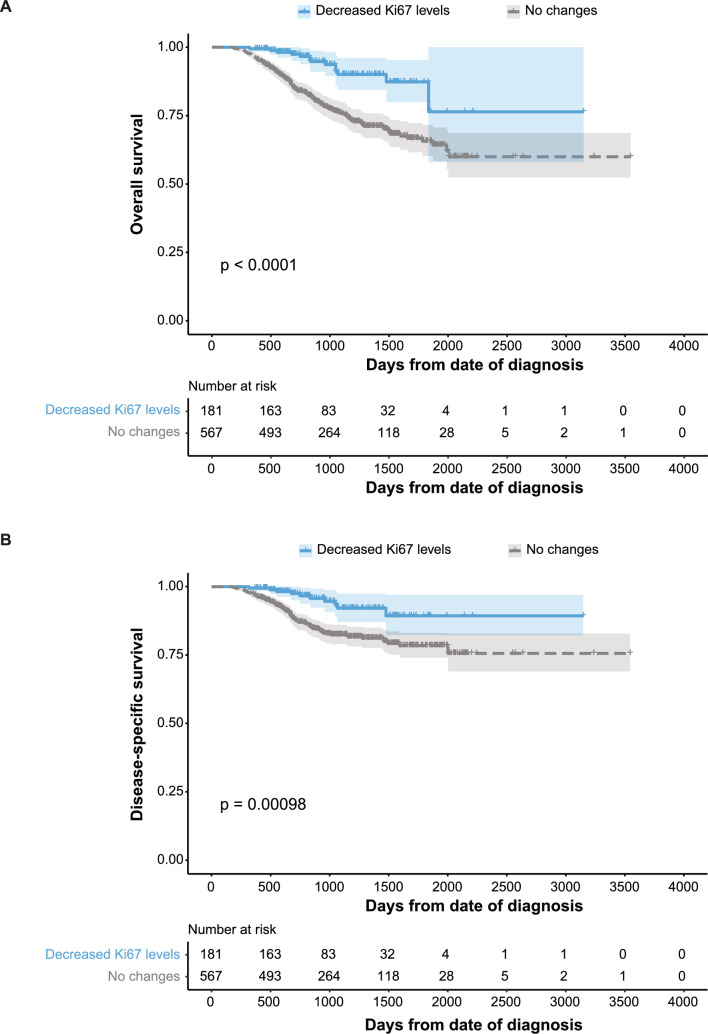
Kaplan–Meier analysis for changes in Ki67 levels after neoadjuvant chemotherapy. Estimates of the probability of **A** overall survival and **B** disease-specific survival based on decreased (blue) or no changes (gray) in Ki67 levels in patients with triple-negative breast cancer (n = 748). Patients with no changes in Ki67 levels post-NACT had the lowest survival probabilities. p-values were calculated using the log-rank test. The x-axes depict days after initial diagnosis and the y-axes depict survival probabilities. Patients with increased Ki67 levels (n = 7) were removed from the analysis

The incidence of death from breast cancer was highest for patients with residual disease; all of the 54 patients achieving pCR were still alive 5 years after initial diagnosis (Fig. 4A). After stratifying the patients with residual disease into the Ki67% groups (no changes and decreased levels; patients with increased levels were removed due to the small sample size), the incidence of death from breast cancer and other causes was highest for patients with no changes in Ki67% (Fig. 4B). Compared to decreased Ki67 levels post-NACT, Kaplan–Meier analysis revealed that the absence of changes in Ki67% was associated with significantly more unfavorable OS and DSS (Fig. 5). Five-year survival rates were significantly lower for patients with tumors showing no changes in Ki67% (OS: 66%; DSS: 78%) compared to patients with decreased Ki67% following treatment (OS: 87%; DSS: 89%). As expected, patients ≥ 75 years of age had the lowest survival probabilities (4-year survival: 76% for decreased Ki67% vs > 87% for patients < 75 years of age; 39% for no changes in Ki67% vs 71% for patients < 75 years of age). Univariable Cox regression analysis demonstrated that RDBN score and no changes in Ki67% were associated with both OS and DSS, while age was only associated with OS (*P* < 0.05, Table 4). After adjusting for age, RDBN score, and changes in Ki67 levels, only RDBN score was an independent predictor of OS and DSS (*P* < 0.001). Changes in Ki67 levels was only found to have an impact on OS in patients between the ages 40 and 74 years (**Supplementary Fig. 1–4**).

**Table 4:** Uni- and multivariable Cox regression analysis for disease-specific and overall survival in TNBC patients with RD

	**Univariable analysis**			
	Disease-specific survival		Overall survival	
Characteristic	HR (95% CI)	*P*	HR (95% CI)	*P*
Age	1.01 (0.99, 1.02)	0.3	1.01 (1.00, 1.03)	**0.022**
RDBN score				
2	Ref		Ref	
3	4.11 (1.46, 11.6)	**0.008**	5.55 (2.22, 13.9)	** < 0.001**
4	21.6 (7.79, 59.9)	** < 0.001**	22.8 (9.18, 56.8)	** < 0.001**
Changes in Ki67 levels				
Decreased levels	Ref		Ref	
No changes	2.86 (1.49, 5.49)	**0.002**	3.26 (1.85, 5.76)	** < 0.001**

	**Multivariable analysis**			
	Disease-specific survival		Overall survival	
Characteristic	HR (95% CI)	*P*	HR (95% CI)	*P*
Age	1.01 (0.99, 1.02)	0.4	1.01 (1.00, 1.02)	0.058
RDBN score				
2	Ref		Ref	
3	3.49 (1.21, 10.1)	**0.021**	4.74 (1.87, 12.0)	**0.001**
4	16.6 (5.80, 48.6)	** < 0.001**	17.6 (6.86, 45.1)	** < 0.001**
Changes in Ki67 levels				
Decreased levels	Ref		Ref	
No changes	1.86 (0.78, 4.45)	0.2	1.90 (0.93, 3.86)	0.077

Statistically significant p-values are highlighted in bold

*CI* Confidence interval, *HR* Hazard ratio, *RD* Residual disease, *RDBN* Residual disease in breast and nodes, *TNBC* Triple-negative breast cancer

## Discussion

This population-based registry study demonstrates that only 20–25% of TNBCs display lower Ki67% and tumor size following neoadjuvant chemotherapy. Remarkably, 71% of TNBCs maintained high Ki67% (> 20% expression) and 75% had no changes in Ki67% post-NACT. The absence of changes in Ki67% was associated with significantly lower OS and DSS, while 5-year survival was not reached for the seven cases with increased Ki67% post-NACT and all 54 patients reaching pCR were still alive 5-years after diagnosis. These findings align with Nishimura et al*.*, which showed the prognostic relevance of Ki67 in breast cancer patients with recurrent disease, revealing the importance of taking the Ki67 index into consideration when making treatment decisions for breast cancer patients, particularly during follow-up and after disease recurrence*.*[16]

Although 69% of the T2-staged tumors decreased in size to T1 post-NACT, 24% of samples retained their T2 classification and 8% increased to T3. According to Lehmann et al*.*, TNBC patients with residual disease post-NACT were more likely to experience recurrence and die of metastatic disease [34]. NACT-treated TNBC patients with residual disease in the breast or axilla at the time of surgery also have a higher risk of locoregional recurrence and lower disease-free survival despite adjuvant radiation therapy [1, 2]. It is well known that hormone receptor-positive breast tumors can change receptor status during treatment [35]. Receptor changes in TNBC cases is less studied.

Notably, changes in breast cancer subtype were observed for 51/754 tumors post-NACT (Luminal A: n = 13; Luminal B/HER2-: n = 25; HER2-positive: n = 13). One tumor also changed from HER2-positive (pre-NACT) to TNBC (post-NACT). Intriguingly, the seven cases with increased Ki67% after treatment were classified as TNBC at baseline and post-NACT. These findings highlight potential challenges with determining breast cancer subtyping using pre-NACT core needle biopsies. As the core biopsy is only a random sample of the primary tumor, the number of biopsies taken may not be representative of the whole tumor or the positioning of different tumor foci, if sampled at all. In Sweden, there are currently no standardized guidelines stipulating how many biopsies should be taken in relation to tumor size. In addition, serial tumor and/or liquid biopsies during NACT could be used as a surrogate for treatment response and have been associated with improved outcome following endocrine treatment [4].

Moreover, this study sheds light on the evolving landscape of breast cancer subtyping, particularly for TNBC, as evidenced by changes in subtype classification post-NACT. Lehmann’s characterization of distinct TNBC subtypes (basal-like 1, basal-like 2, mesenchymal, and luminal androgen receptor) with varying response to chemo- and radiation therapy underscores the complexity of TNBC heterogeneity. [10, 34, 36, 37] [38, 39] Furthermore, choices whether to use immunotherapy and other treatment options could be impacted by the lack of immune cells in certain TNBC subtypes (e.g., mesenchymal TNBCs) [37], as well as, the abundance of treatment-resistant breast cancer stem cells in basal-like 2 and mesenchymal tumors [40]. Despite the potential implications for treatment decisions, TNBC subtyping has not found its way into routine clinical practice, limiting therapeutic options to conventional chemotherapy often with the intention of shrinking the tumor prior to deciding on the choice of surgery. If the given chemotherapy does not have the desired effect, the costs to the patient (i.e., potential side effects) and healthcare system ought to be seen as too high.

Ki67 is an excellent marker of proliferation, providing valuable insights into prognosis and treatment response across various breast cancer subtypes [41, 42]. The International Ki67 in Breast Cancer Working Group recently proposed Ki67 ≤ 5% (low aggressive tumors) or ≥ 30% (high aggressive tumors) as reliable prognostic indicators for small T1-2 breast tumors (with or without metastatic spread to the axillary lymph nodes) [43]. However, challenges persist in analytical validity, including the lack of standardized cutoff values and reproducibility due to the subjective evaluation (“eye balling”) of Ki67 in hotspots, hindering its seamless integration into routine clinical practice [25]. To address these challenges, the integration of artificial intelligence (AI) in pathology offers a promising solution. AI technologies, particularly in the field of image analysis, have exhibited the potential to automate the assessment of Ki67 expression, establishing standardized cutoff values and enhancing the accuracy of Ki67 assessment in the entire tumor area to distinguish normal tissue DCIS from invasive tissue. This advancement contributes to fostering greater consistency in results across diverse laboratories. This innovative approach could enhance the seamless integration of Ki67 into routine clinical practice by mitigating the current challenges associated with its analytical validity.

A recent study suggests that changes in Ki67 expression after neoadjuvant endocrine therapy are predictive of pCR, recurrence-free survival, and overall survival in patients with hormone receptor-positive breast cancer [41]. In hormone receptor-positive breast cancer, Ki67 serves as a guide for treatment decisions, influencing the choice of endocrine therapy and chemotherapy [44]. For patients with TNBC, Ki67's role primarily extends to predicting treatment response rather than offering prognostic information [41]. Penault et al*.* suggests that the pre-NACT Ki67 index is predictive of treatment response, while the post-NACT Ki67 index has prognostic value [44]. Finkelman and colleagues showed that higher pre-treatment Ki67 values are associated with a greater probability of pCR and worse survival [4]. Moreover, increased Ki67, but not pCR, is a poor prognostic marker in non-responders to NACT [4, 5]. In the present study, pCR was associated with favorable clinical outcome (100% survival), while patients with RDBN score 4 had the lowest 5-year survival rates. Interestingly, the pCR group also had slightly higher Ki67 levels at baseline, suggesting the influence of other factors on treatment response and patient survival. However, the pCR group was relatively small, in part, due to the high degree of missing data (564/1777 patients) regarding the presence of invasive disease (invasive carcinoma with or without cancer in situ, or solely cancer in situ) in the resection specimen. Indeed, though pathologic response rates also vary depending on the TNBC subtype (the basal-like 2 and luminal androgen receptor TNBC subtypes have the lowest pCR rates), this information was not available [34].

By using multiple national registries with high validity, high data completeness, and mandatory data reporting, we were able to compile a relatively large cohort of TNBC patients treated with NACT from around Sweden [45]. Data linkage between the different registries was done using the personal identification number, which is given to all Swedish residents. We also used a Charlson Comorbidity Index adapted for use with Swedish registry-based data [26]. However, quite a few patients were excluded from the study due to incomplete data for Ki67 status at baseline and/or post-NACT, raising the concern of population bias. Although data imputation was not an option in this case because more than 5% of the Ki67 values at baseline and/or after treatment were missing, we still believe the data are relevant. Therefore, no patients diagnosed before 2013 were included in the study. To associate changes in Ki67% with treatment response, it would have been useful if we had more information regarding Residual Cancer Burden, TNBC subtype, and whether immunotherapy had been administered. Furthermore, the univariable analyses should be taken with caution due to the number of patients in the Ki67 groups. In the multivariable Cox regression analysis, the importance of changes in Ki67% vanishes because the primary factor is the presence of residual cancer.

In the present study, we show that most patients treated with NACT still have TNBCs with high Ki67 expression and no change in Ki67% after treatment. From the perspective of the patient and healthcare system, these findings demonstrate that patients with TNBC should be offered more individualized treatment, specifically tailored for those that would benefit the most from neoadjuvant chemotherapy. Further studies should delve into the potential of novel tools for subclassifying TNBC based on biology, clinical outcome, and treatment response (e.g., TNBC subtyping and tumor-infiltrating leukocytes). Recognizing the dynamic nature of tumor characteristics during treatment, continuous monitoring through additional tumor sampling or liquid biopsies becomes imperative to tailor interventions, avoid adverse clinical outcomes, and enhance treatment efficacy. Moving forward, efforts should focus on evaluating the utility of the Ki67 index as a surrogate marker for OS in patients with TNBC. Additionally, exploring circulating tumor DNA at multiple intervals during treatment holds promise for identifying non-responders at an early stage. This collective approach, integrating comprehensive subclassification tools and dynamic monitoring, lays the foundation for a more nuanced and individualized treatment landscape for TNBC patients, promising improved outcomes and minimized adverse effects.

## Supplementary Information

Below is the link to the electronic supplementary material.

Supplementary file1 (PDF 565 KB)

Supplementary file2 (PDF 568 KB)

Supplementary file3 (PDF 494 KB)

## Data Availability

No datasets were generated or analyzed during the current study request to the corresponding author.
