# Supplementary material for: The prognostic value of changes in Ki67 following neoadjuvant chemotherapy in residual triple-negative breast cancer: a Swedish nationwide registry-based study
Source: Breast Cancer Res Treat. 2025 Jan 12;210(3):719–36. doi: 10.1007/s10549-025-07610-z (PMC11953087; doi:10.1007/s10549-025-07610-z)
Supplement: Supplementary file 2 — Supplementary file2 (PDF 568 KB) [file 10549_2025_7610_MOESM2_ESM.pdf]

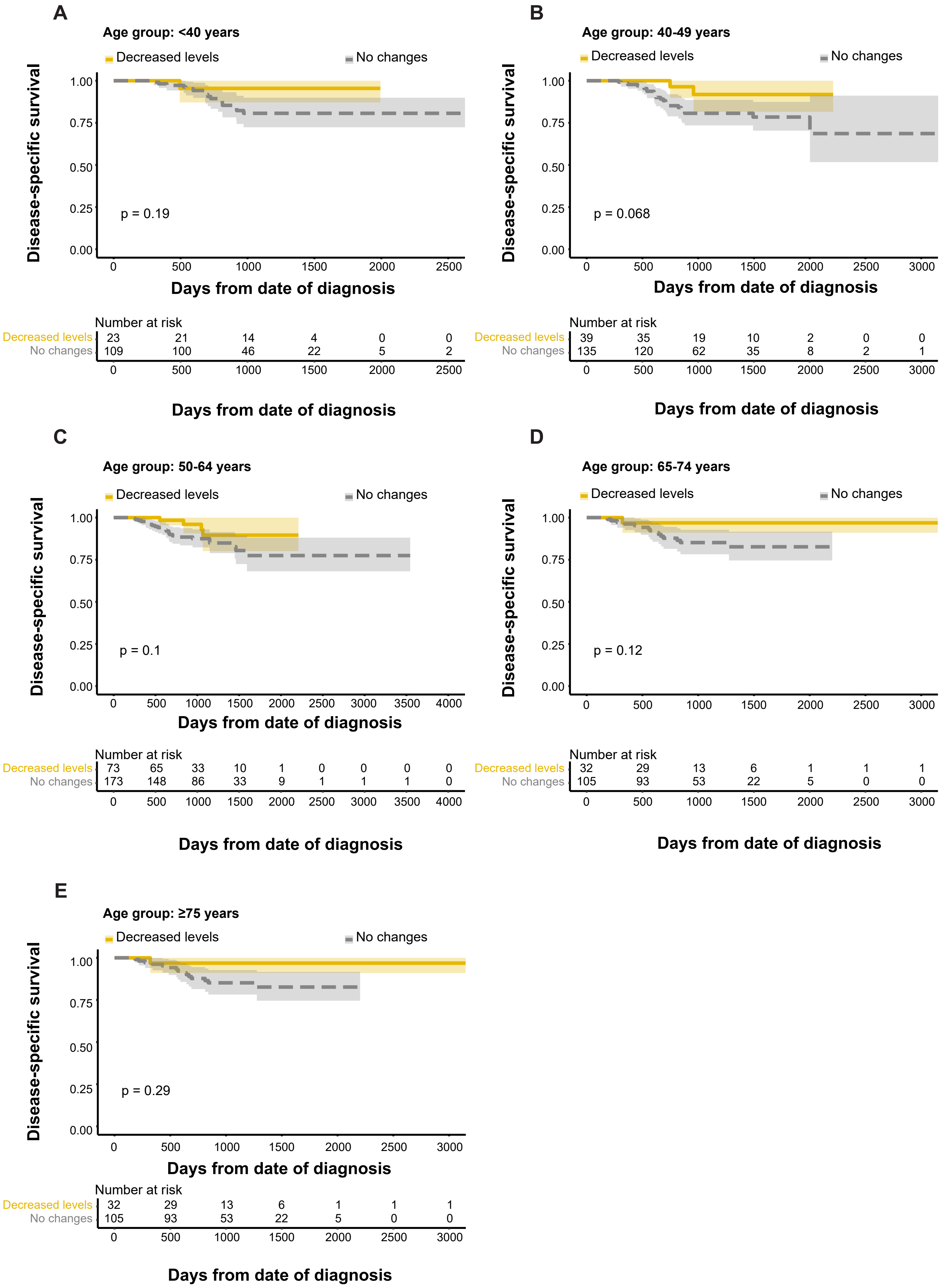
A
Age group: <40 years
Decreased levels
No changes
Disease-specific survival
p = 0.19
Days from date of diagnosis
Number at risk
Decreased levels 23 21 14 4 0 0
No changes 109 100 46 22 5 2
0 500 1000 1500 2000 2500
B
Age group: 40-49 years
Decreased levels
No changes
Disease-specific survival
p = 0.068
Days from date of diagnosis
Number at risk
Decreased levels 39 35 19 10 2 0 0
No changes 135 120 62 35 8 2 1
0 500 1000 1500 2000 2500 3000
C
Age group: 50-64 years
Decreased levels
No changes
Disease-specific survival
p = 0.1
Days from date of diagnosis
Number at risk
Decreased levels 73 65 33 10 1 0 0 0 0
No changes 173 148 86 33 9 1 1 1 0
0 500 1000 1500 2000 2500 3000 3500 4000
D
Age group: 65-74 years
Decreased levels
No changes
Disease-specific survival
p = 0.12
Days from date of diagnosis
Number at risk
Decreased levels 32 29 13 6 1 1 1
No changes 105 93 53 22 5 0 0
0 500 1000 1500 2000 2500 3000
E
Age group: ≥75 years
Decreased levels
No changes
Disease-specific survival
p = 0.29
Days from date of diagnosis
Number at risk
Decreased levels 32 29 13 6 1 1 1
No changes 105 93 53 22 5 0 0
0 500 1000 1500 2000 2500 3000
