# Supplementary material for: The prognostic value of changes in Ki67 following neoadjuvant chemotherapy in residual triple-negative breast cancer: a Swedish nationwide registry-based study
Source: Breast Cancer Res Treat. 2025 Jan 12;210(3):719–36. doi: 10.1007/s10549-025-07610-z (PMC11953087; doi:10.1007/s10549-025-07610-z)
Supplement: Supplementary file 3 — Supplementary file3 (PDF 494 KB) [file 10549_2025_7610_MOESM3_ESM.pdf]

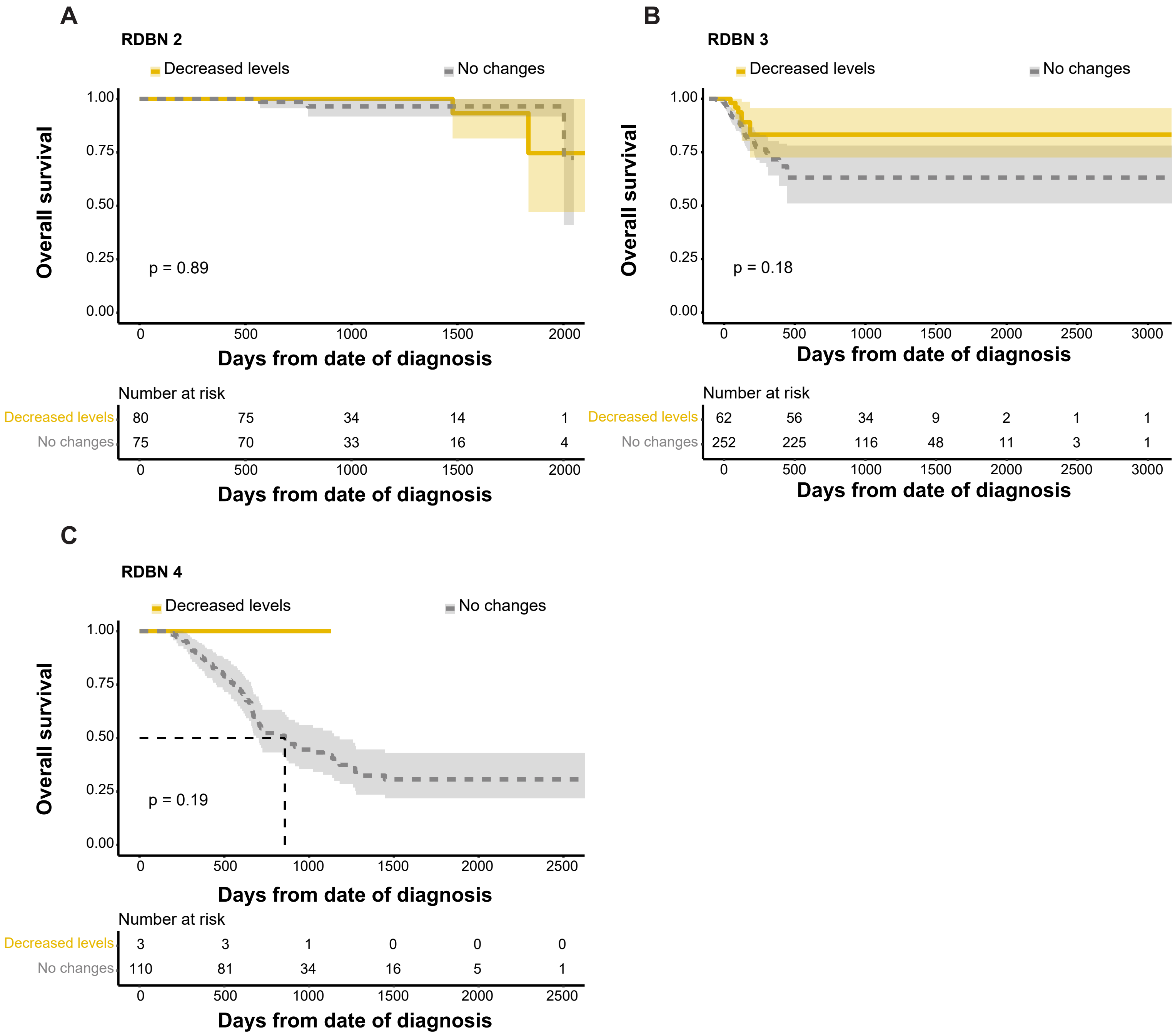
A
RDBN 2
Decreased levels
No changes
Overall survival
1.00
0.75
0.50
0.25
0.00
p = 0.89
0
500
1000
1500
2000
Days from date of diagnosis
Number at risk
Decreased levels 80 75 34 14 1
No changes 75 70 33 16 4
0
500
1000
1500
2000
Days from date of diagnosis
B
RDBN 3
Decreased levels
No changes
Overall survival
1.00
0.75
0.50
0.25
0.00
p = 0.18
0
500
1000
1500
2000
2500
3000
Days from date of diagnosis
Number at risk
Decreased levels 62 56 34 9 2 1 1
No changes 252 225 116 48 11 3 1
0
500
1000
1500
2000
2500
3000
Days from date of diagnosis
C
RDBN 4
Decreased levels
No changes
Overall survival
1.00
0.75
0.50
0.25
0.00
p = 0.19
0
500
1000
1500
2000
2500
Days from date of diagnosis
Number at risk
Decreased levels 3 3 1 0 0 0
No changes 110 81 34 16 5 1
0
500
1000
1500
2000
2500
Days from date of diagnosis
